# PITDB: a database of translated genomic elements

**DOI:** 10.1093/nar/gkx906

**Published:** 2017-10-09

**Authors:** Shyamasree Saha, Eleni A Chatzimichali, David A Matthews, Conrad Bessant

**Affiliations:** School of Biological and Chemical Sciences, Queen Mary University of London, Mile End, London E1 4NS, UK; School of Cellular and Molecular Medicine, University of Bristol, University Walk, Bristol BS8 1TD, UK; Centre for Computational Biology, Life Science Institute, Queen Mary University of London, Mile End, London E1 4NS, UK

## Abstract

PITDB is a freely available database of translated genomic elements (TGEs) that have been observed in PIT (proteomics informed by transcriptomics) experiments. In PIT, a sample is analyzed using both RNA-seq transcriptomics and proteomic mass spectrometry. Transcripts assembled from RNA-seq reads are used to create a library of sample-specific amino acid sequences against which the acquired mass spectra are searched, permitting detection of any TGE, not just those in canonical proteome databases. At the time of writing, PITDB contains over 74 000 distinct TGEs from four species, supported by more than 600 000 peptide spectrum matches. The database, accessible via http://pitdb.org, provides supporting evidence for each TGE, often from multiple experiments and an indication of the confidence in the TGE’s observation and its type, ranging from known protein (exact match to a UniProt protein sequence), through multiple types of protein variant including various splice isoforms, to a putative novel molecule. PITDB’s modern web interface allows TGEs to be viewed individually or by species or experiment, and downloaded for further analysis. PITDB is for bench scientists seeking to share their PIT results, for researchers investigating novel genome products in model organisms and for those wishing to construct proteomes for lesser studied species.

## INTRODUCTION

Annotation of genomes is a significant endeavor in modern biology, as we seek a comprehensive picture of the many distinct elements each genome contains and try to determine the role that these elements play. After many years of research in the area it is tempting to assume that predicting which genomic elements code for proteins is a solved problem, but recent research has shown this is not the case. Even in *Homo sapiens*, experimental studies have suggested that widely accepted protein coding regions are not seen to express proteins (1,2), while supposedly non-coding elements such as pseudogenes and so-called non-coding RNAs (ncRNAs) are in fact translated (1–4). Furthermore, most human genes express multiple protein isoforms through alternate splicing, and novel genomic products have been observed such as fusion proteins (5–9) and short open reading frames (sORFs) (3,10,11). Expression of these products is currently difficult to predict computationally. In non-model organisms our understanding is worse still, with no reliable catalogue of the proteome available for many important species. For example, the notable disease vector black flying fox *(Pteropus alecto*) has just two experimentally confirmed proteins in UniProt.

The advent of RNA-seq (12,13) transcriptomics has gone some way toward solving the genome annotation problem, by allowing high-throughput open and unbiased sequencing of transcripts that can be mapped back to the genome. *De novo* transcript assembly tools such as Trinity (14), and emerging long read sequencing methods such as PacBio (15) even allow full length transcripts to be sequenced without a reference genome assembly. However, the presence of a transcript does not by itself tell us whether that transcript is translated into an amino acid chain. For that, we have previously developed the PIT (proteomics informed by transcriptomics) methodology (16). PIT uses RNA-seq data to generate species-blind sample-specific search databases for liquid chromatography tandem mass spectrometry (LC-MS/MS) shotgun proteomics, thereby facilitating the unbiased identification of translated genomic elements (TGEs) even in the absence of a reference proteome. This contrasts with traditional proteomics, where the proteomic mass spectra are searched against standard canonical proteomes, prohibiting the discovery of novel TGEs. We use the term TGE because these molecules are amino acid chains derived from the genome but we cannot guarantee that they are all viable proteins, although in practice the vast majority of TGEs are indeed proteins.

To facilitate the complex process of analyzing data from PIT experiments, we have implemented workflows for such analysis on a dedicated publicly available Galaxy (17) server called GIO (Galaxy Integrated Omics) (18) (gio.sbcs.qmul.ac.uk). These workflows allow rapid and repeatable analysis of PIT data with results produced in a uniform format. The availability of these workflows has led to an increasing uptake of the PIT approach, leading in turn to the creation of more matched RNA-seq and LC-MS/MS datasets. These experiments are generally intended to answer specific biological questions and data from these has been analyzed with those questions in mind, but there is a clear benefit to bringing these datasets together as they contain a substantial amount of information about a diverse range of TGEs, which can be integrated and mined. While the output of our PIT workflows is very comprehensive and uniform in format (we offer tabular output, GFF3 genome annotation files and links back to the workflows used and the original data) to date there has been no structured repository for the sharing and integration of these results. Sharing and comparing data is essential if we are to build confidence in potential novel findings such as novel protein isoforms and other interesting TGEs. It can also help to refine genome annotation in model organisms and accelerate the annotation of recently sequenced genomes from non-model species.

Here we present a data sharing solution in the form of PITDB, a web accessible database of PIT results. At the heart of this database are the TGEs, each of which is supported by evidence at the mRNA and peptide level and has associated metadata about the sample(s) in which the TGE was observed. Many of the TGEs have been observed in multiple samples, some from multiple species, and the evidence for individual TGEs is strengthened as more experimental data is added.

At the most basic level, PITDB can be used to share the results of a PIT experiment, in support of a publication. While repositories exist for transcriptomic and proteomic data, PITDB is currently the only database that brings these data types together in a fully integrated way. Wider applications of PITDB include the identification of novel TGEs, including novel isoforms of known proteins, in model organisms such as human. Although the chance of finding novel TGEs in well-studied species is small, such TGEs are likely to be of great interest. For lesser studied organisms PITDB provides a rapid route to a draft proteome. This proteome can be analyzed computationally, or can be used as a search database for further proteomics experiments.

## IDENTIFICATION AND CLASSIFICATION OF TGEs

**Figure 1.**
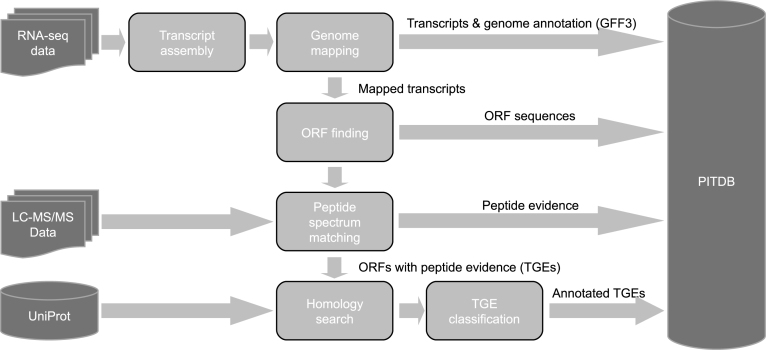
Simplified schematic showing how the PIT workflow populates PITDB. First, TGEs are found by *de novo* assembling transcripts from RNA-seq data, mapping these against a genome, then searching MS/MS data from the same sample against ORFs generated from the transcripts. ORFs with peptide evidence (TGEs) are then BLASTed against protein sequences from UniProt to classify them as known, novel, isoform, etc. and assess the level of confidence in that classification using the factors shown in Table 1. All key results generated during the process are deposited in the integrated PITDB database, which can be accessed via the web.

In every experiment covered by PITDB, both RNA-seq and LC-MS/MS data were collected from the same sample. PITDB is populated with TGEs identified from this data using an enhanced version of our previously published genome-guided PIT workflow (18), a high level overview of which is shown in Figure 1. The first step in this workflow is *de novo* assembly of the RNA-seq short reads into transcripts using Trinity (14). These transcripts are then passed on to the Program to Assemble Spliced Alignments (PASA) (19), which first runs the SeqClean (https://sourceforge.net/projects/seqclean/) utility to identify and remove poly(A) tails, trim vectors and remove low quality sequences. PASA maps the remaining transcripts to a reference genome using a spliced alignment process that infers the intron–exon structure of the parent gene. PASA assembles clusters of overlapping transcript alignments (overlapping transcripts that have the exact same gene structure in the overlapping region) into maximal alignment assemblies that are partial/prematurely ended assemblies of Trinity. By doing this it reduces the number of incomplete ORFs (ORFs that are missing either or both the start and end codons) and duplicate transcripts, minimizing search space in the later peptide identification step. Any transcripts that do not map to the selected genome assembly (e.g. from viruses that may be present in the sample) are discarded at this stage. Transdecoder (20) is then used to find ORFs within the PASA assembled transcripts. ORFs smaller than 11 AAs long and encapsulated inside other ORFs are filtered out. Transdecoder also produces GFF3 and BED files for the predicted ORFs, which are used in PITDB to show genomic context. The final list of ORFs (augmented with sequences of common contaminant proteins to avoid false identifications) is then used to identify peptide spectra from the corresponding LC-MS/MS data. MSGF+ (21) was used for peptide spectrum matching, and mzIdentML-lib (22) for post processing and protein inference. Search parameters (e.g. fixed and variable modifications, mass tolerance etc.) for MSGF+ were set according to the proteomics protocol used in each experiment. A target-decoy search strategy was employed, with a 1% peptide spectrum match (PSM) level false discovery rate cut-off applied throughout. Identified TGEs with less than two peptides were removed, giving a final list of TGEs for upload to PITDB.

At this point the only identifying information we have about each TGE is its amino acid sequence. Further processing is needed to determine whether each TGE is a known protein, a variant of an existing protein, or something novel. The first step in this process is to BLAST each TGE sequence against the UniProt complete proteome (including both SwissProt and TrEMBL sequences, and isoforms where available) for the species being studied. For the purposes of this comparison, a BLAST e-value below 1 × 10^−30^ is taken to indicate a match between identified sequences as this is widely regarded as indicating strong homology between proteins. We classify the TGE based on the type and strength of alignment it has against the UniProt proteins. Identified TGEs with an exact match (100% sequence identity) to a UniProt protein are labeled as known proteins and the accession number of the matching protein recorded. A TGE is labeled as a known protein variant when the BLAST e-value threshold is met and the alignment covers the full length of the TGE and the UniProt protein but it is not an exact match. The alignment may include single or multiple amino acid differences, insertions or deletions. Some TGE sequences map to a UniProt protein with the required *e*-value but may not cover the whole length of the protein, or may extend beyond the start or end of the protein. The alignments may or may not have variations as well. These TGEs are classified as potential novel isoforms of the protein. TGEs that do not map to any UniProt protein with a BLAST *e*-value below the 1 × 10^−30^ threshold are classed as novel TGEs. These TGEs may be proteins that have not previously been observed or predicted for the species under study, or a more exotic molecule such as a sORF, translated ncRNA, or fusion protein. Further analysis is needed to determine exactly what they may be.

The pipeline for TGE identification and classification has been made publicly available so that researchers can apply it to their own data, to generate PIT results suitable for submission to PITDB. Submission instructions can be found on the web site.

## DATABASE ORGANIZATION AND CONTENT

The fundamental object within PITDB is the TGE. Each TGE has a unique amino acid sequence and accession number (e.g. TGE0000273). Through the aforementioned classification procedure each TGE is also assigned a class, and a UniProt accession number if a homologous protein is found. Every TGE is derived from one or more TGE observations, each of which is in turn supported by experimental evidence from both transcriptomics (transcripts assembled from RNA-seq reads) and proteomics (PSMs). The evidence for each observation is stored within the database and used to determine the level of confidence in the observation of all protein variants. Confidence is represented in the PITDB user interface as a star rating according to the scheme shown in Table 1. Each observation is from a specific species and a specific sample, which is described by metadata including the experiment to which the sample belongs. In turn, experimental metadata summarizes the source of the sample, including the publication to which it relates.

**Table 1.** Scheme used to assign confidence ratings to TGE observations that BLAST suggests are variants of known proteins

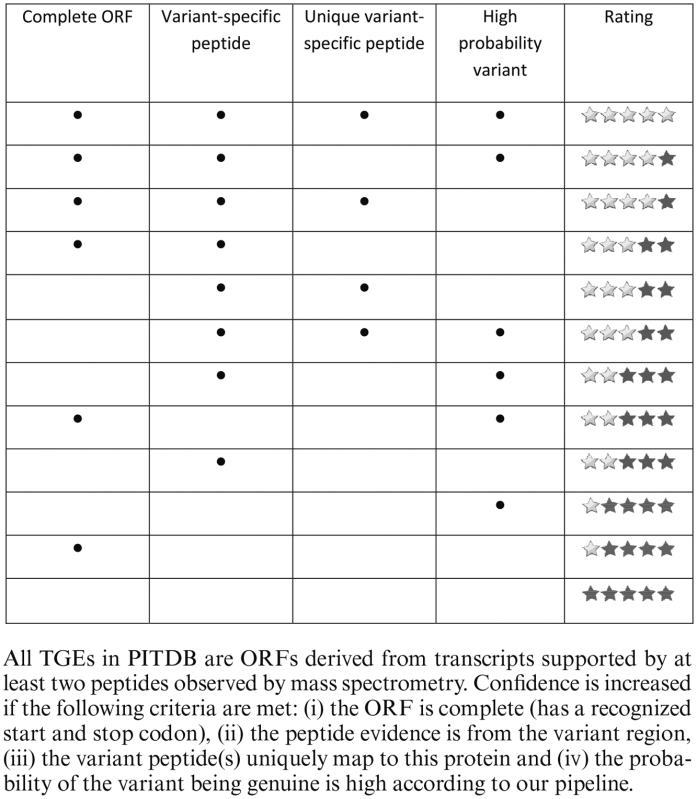

At the time of writing, PITDB contains over 74,000 distinct TGEs from four species (*H. sapiens, Mus musculus, Aedes aegypti, P. alecto*). A summary of these TGEs is shown in Table 2. The total number of TGEs varies by species according to the type and number of experiments captured by PITDB for that species. For example, the total number of human TGEs is relatively low (12,877) because the majority of human samples in PITDB are from the extracellular matrix. *Pteropus alecto* and *M. musculus* have approximately double the number of TGEs because data from multiple whole cell lysate samples from these species is present in the database. PITDB contains TGEs classed as novel (i.e. insufficient homology with any UniProt protein from the species under study or no mapping) for all four species. As may be expected, human has by far the lowest number of novel TGEs (119, none of which have unique peptide evidence) as it has a very well documented proteome. Conversely, *P. alecto* has 1,066 novel TGEs thanks to its relatively incomplete UniProt proteome.

**Table 2.** Overview of PITDB data content at the time of writing

Species	Samples	Known proteins				High confidence novel TGEs (3★ or more)
		Exact match to UniProt protein or isoform	UniProt protein with polymorphisms	Other isoforms	High confidence isoforms (3★ or more)	
*H. sapiens*	31	3,008	254	9,615	77	2
*P. alecto*	10	1,008	303	29,234	1,767	331
*M. musculus*	8	2,384	464	21,534	123	20
*A. aegypti*	1	2,017	101	3,137	540	0

We have TGEs from four species including two well-studied species (human and mouse) and two without a well-established proteome (*P. alecto* and *A. aegypti*). TGEs are categorized into 19 classes: known protein, known protein with variation, 16 distinct types of novel isoform and novel based on their BLAST alignments to reference proteomes of the species under study. A small percentage of identified TGEs have variations such as single amino acid polymorphisms (SAPs), multiple amino acid alterations (ALT), insertions and deletions. Among the isoforms of known proteins, a large proportion of TGEs show partial mapping to an existing protein with a longer or shorter sequence.

### Web interface

The data within PITDB can be accessed via the web interface by browsing by experiment and sample, or through one of six views that are accessed via a simple search box. One of these is the experiment view, which provides an overview of a specified experiment. This overview includes summary statistics such as the number of samples in the experiment and the total number of TGEs observed, a description of the experiment and graphical and tabular overviews of the observed TGEs. This experiment view can be used to share the results of a particular published experiment, by citing the PITDB experiment accession number (e.g. EXP000001) in the publication. The sample view provides similar functionality at the individual sample level.

**Figure 2.**
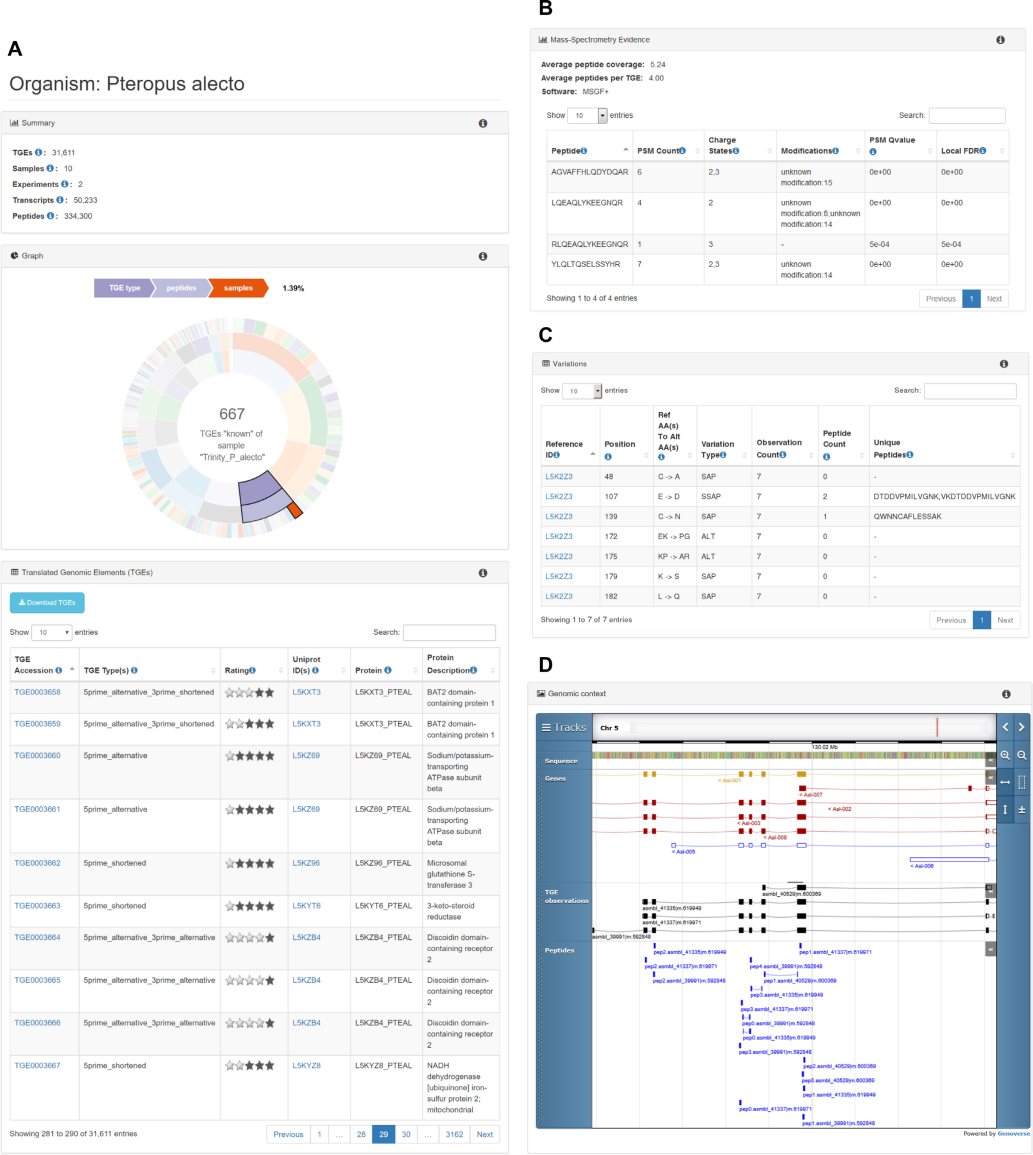
Examples of some key elements of PITDB’s user interface, including (**A**) the organism summary page for *Pteropus alecto* showing the total number of TGEs etc. in numerical and graphical form and providing access to TGEs via an interactive table; (**B**) summary of mass spectrometry evidence for TGE0070846 (a potential novel isoform of human Tetratricopeptide repeat protein 9C); (**C**) variations in sequence found between TGE0000273 and *P. alecto* Ras-related protein Rap-1A protein (UniProt accession L5K2Z3); (**D**) the genomic context of TGE and peptide observations associated with mouse protein E0CY49.

The species view provides access to PITDB’s content related to a selected species. It can be considered an experimentally derived, though almost certainly incomplete, proteome of the species in question. This view (see Figure 2A for an example) shows a summary of the information that PITDB holds about that species, including the total number of TGEs observed. The full list of TGEs is shown in an interactive table, which can be searched and sorted to find TGEs of interest. TGEs can be downloaded in tabular (CSV) format, or as FASTA files, for further analysis by clicking the download button above the table. Similar functionality is provided for other tables throughout PITDB. The FASTA file may be used in the construction of a database against which to search proteomic mass spectrometry data in future experiments.

The TGE view is accessed by clicking on a TGE in a table, or by searching for a specific TGE by either its accession number, or by a full or partial sequence. This view summarizes everything that PITDB knows about a particular TGE, including the species in which it has been observed, UniProt proteins with which it shares homology, and details of the individual observations including the transcript sequence underlying the observation and details of the mass spectrometry evidence (PSMs and their *q*-values—see Figure 2B). If appropriate, amino acid differences between the observed TGE and homologous proteins are also shown in a variations tables (Figure 2C).

The protein view shows all TGEs that are homologous with a known UniProt protein. It is necessarily only available for proteins from species with well annotated genomes and proteins in UniProt. However, it includes both SwissProt and TrEMBL proteins so can be used to confirm the existence of a protein that had previously only been computationally predicted. PITDB TGEs relating to the specified protein are shown in their genomic context (using the GFF files produced by Transdecoder), together with known genome annotations from Ensembl and PIT peptide evidence (see Figure 2D). Boundary crossing peptides that define introns can clearly be seen—a good example of transcriptomic information being reinforced by proteomic data. Clicking on a feature in the genome browser brings up a box containing further details, for example the q-value of the identification in the case of peptides.

Protein views can also be accessed via the gene search functionality. Here, the user enters a gene symbol (e.g. COL6A3) and is then presented with a list of any protein products of that gene for which there is evidence in PITDB. Clicking on one of the proteins listed leads directly to the relevant protein view.

## DISCUSSION

PITDB is a unique repository of experimentally observed TGEs, built on data from both RNA-seq and LC-MS/MS performed on the same samples. There is much work to do in analyzing the content of this database, for example to investigate novel protein isoforms and TGEs. PITDB’s architecture is eminently scalable and we plan to continue adding more PIT results, which will increase both the breadth of species covered and the strength of evidence underpinning individual TGEs. We have also identified a number of additional features which would increase the research value of PITDB. First among these is the addition of support for quantitative data, as RNA-seq is inherently quantitative and SILAC (23–26) and TMT (27) protocols are often used to provide protein abundance information in PIT experiments. It would also be useful to extend the TGE classification workflow to provide a more fine-grained classification of novel TGEs, by automatically searching against databases of known sORFs and ncRNAs for example.
